# The Role of Cognitive and Affective Empathy in Spouses' Support Interactions: An Observational Study

**DOI:** 10.1371/journal.pone.0149944

**Published:** 2016-02-24

**Authors:** Lesley Verhofstadt, Inge Devoldre, Ann Buysse, Michael Stevens, Céline Hinnekens, William Ickes, Mark Davis

**Affiliations:** 1 Department of Experimental Clinical and Health Psychology, Ghent University, Ghent, Belgium; 2 Department of Experimental Psychology, Ghent University, Ghent, Belgium; 3 Department of Psychology, University of Texas, Arlington, Texas, United States of America; 4 Department of Psychology, Eckerd College, St. Petersburg, Florida, United States of America; Oregon Health and Science University, UNITED STATES

## Abstract

The present study examined how support providers’ empathic dispositions (dispositional perspective taking, empathic concern, and personal distress) as well as their situational empathic reactions (interaction-based perspective taking, empathic concern, and personal distress) relate to the provision of spousal support during observed support interactions. Forty-five committed couples provided questionnaire data and participated in two ten-minute social support interactions designed to assess behaviors when partners are offering and soliciting social support. A video-review task was used to assess situational forms of perspective taking (e.g., empathic accuracy), empathic concern and personal distress. Data were analyzed by means of the multi-level Actor-Partner Interdependence Model. Results revealed that providers scoring higher on affective empathy (i.e., dispositional empathic concern), provided lower levels of negative support. In addition, for male partners, scoring higher on cognitive empathy (i.e., situational perspective taking) was related to lower levels of negative support provision. For both partners, higher scores on cognitive empathy (i.e., situational perspective taking) correlated with more instrumental support provision. Male providers scoring higher on affective empathy (i.e., situational personal distress) provided higher levels of instrumental support. Dispositional perspective taking was related to higher scores on emotional support provision for male providers. The current study furthers our insight into the empathy-support link, by revealing differential effects (a) for men and women, (b) of both cognitive and affective empathy, and (c) of dispositional as well as situational empathy, on different types of support provision.

## Introduction

Social support refers to the ability of relationship partners, such as spouses, to help each other cope with personal difficulties [1]. Overall, one can distinguish positive from negative forms of support provision [2]. Positive support provision can take the form of *emotional support behaviors* such as reassuring, consoling, and encouraging the support seeker (i.e., emotional support provision); and *instrumental support behaviors* such as giving advice, offering assistance, or making specific suggestions to the support seeker (i.e., instrumental support provision). On the other hand, so-called *negative support provision* includes behavior that is perceived as unhelpful and sometimes even not intended to be helpful, such as offering an analysis of the problem without considering the support seeker’s view, discounting the importance of the support seeker’s problem, giving useless advice, and even criticizing and blaming the support-seeking spouse for the problem under discussion [1].

Empirical studies have consistently shown that the way spouses provide everyday support to one another plays a central role in relationship functioning [3]. Greater provision of positive support from the partner (i.e., emotional and instrumental support) enables the support seeker to explore options and progress towards goals in ways that enhance both relationship and personal well-being [4]. On the other hand, there is growing evidence that negative forms of support can actually be harmful to the support recipient and to the relationship [5][6].

The giving and taking of support within a couple is a multi-faceted interpersonal interaction that involves a complex sequence of steps [7]. Pearlin and McCall [8] have delineated the sequential stages that occur in support transactions between spouses. In the first stage, the potential support provider must perceive that his/her partner has a problem and that offering support to the partner is a possible response. In the second stage, the potential provider must evaluate the situation to determine whether or not to make an offer of support, and if so, what form it should take. At this stage, the support provider makes judgements about the extent of the support-seeker’s need, the kind of support that could be provided, and the likelihood that the support will be successful. Finally, in the third stage, actual support is provided (or not), based on the analysis occurring at the second stage [9].

It is commonly assumed that the manner in which these three stages of support transactions are navigated will have important consequences for the success or failure of the support episode [10]. Previous work [11]-[13] noted that potential support providers who try to understand the needs and assess the coping resources of their support-seeking partner are often faced with *incomplete knowledge* about all of these aspects, resulting in a variety of difficulties.

First, partners in distress may not always actively seek support from the potential provider [7]. Second, even in those cases in which spouses do seek help, their communication may take an indirect form. For example, they may hint or complain, rather than verbalize their need for support more directly [14]. Third, a distressed partner’s tactics for activating social support are often nonverbal in nature (e.g., taking the form of sighing, fidgeting, or displaying “helpless” facial expressions) and as a result are often somewhat ambiguous [15]. Part of the problem is that people often assume that their partners should be able to interpret such subtle indications without being told explicitly what they mean [10]. In consequence, potential support providers are faced with making difficult decisions that are potentially based on incomplete knowledge about their partner’s desire or need for assistance, the kind of support that is needed, and the partner’s personal resources for dealing with the challenge.

In the present article, we assume that the extent to which a potential support provider “tunes in to” the support-seeking partner’s internal states—both cognitively and affectively—will play a cardinal role in support provision. More specifically, we suggest that spousal support provision is affected by two processes: *cognitive and affective empathy*. Empathy can be broadly defined as the tendency to react to other people’s observed experiences [16]. It includes both *knowing* what the other person thinks and feels (i.e., cognitive empathy [17]) and *feeling* something of what the other person feels (i.e., affective empathy [18][19]).

### Cognitive empathy

The cognitive component of empathy involves understanding what the other person thinks and feels. One approach for measuring this component, proposed by Davis [20], is to use a self-report questionnaire to assess the trait of *perspective-taking*. A second approach for measuring this component, proposed by Ickes, Stinson, Bissonnette, and Garcia [21], is to use a video-cued procedure to assess situational perspective taking (i.e., empathic accuracy) as a performance measure.

The first approach presumes that people vary in the degree to which they are generally predisposed to adopt the psychological perspective of others. The second approach presumes that people’s success in “reading” other people’s current thoughts and feelings can be measured directly. Instead of using a trait measure to assess people’s perceptions of the degree to which they are generally disposed to take other people’s perspectives, the empathic accuracy approach requires people to infer another person’s successive thoughts and feelings during actual interactions and then measures the actual degree of inferential accuracy that was achieved [22][23].

Both of these measures—the more global and”distal” trait measure of perspective-taking and the more situational and the “proximal” performance measure of perspective taking (i.e., empathic accuracy)—have been related in previous studies to support provision in romantic relationships. In a survey study, Devoldre and colleagues [24] found that the dispositional tendency to take the perspective of others was positively correlated with providing more positive partner support (emotional and instrumental), but negatively correlated with offering negative support. In laboratory-based couple interaction studies, providers’ level of empathic accuracy (i.e., situational perspective taking) proved to be positively correlated with greater levels of instrumental support [11][12]. However, when the support provider was unsuccessful in “reading” the contents of the partner’s thoughts and feelings, more negative support was provided. These empirical precedents led us to include both the trait measure of perspective-taking and the performance measure of situational perspective-taking as our cognitive-empathy predictors of spousal support in the present investigation.

### Affective empathy

In addition to cognitive empathy, an *affective component* of empathy exists. The affective position views empathy as an observer’s emotional response to another person’s experiences, although this emotional response can take various forms. Some investigators have argued that empathy consists of the specific emotional response of compassion/concern for the other person [25]; this construct is also referred to as *empathic concern* [16] or sympathy [26]. For others, empathy is defined as the observer experiencing the same (or similar) emotions as the target (e.g., [27])—a response sometimes referred to as parallel empathy [20] or emotional contagion [28]. The latter then refers to the observer experiencing feelings of distress (i.e., *personal distress*) when witnessing the negative experiences of others.

Research using Davis’s Interpersonal Reactivity Index (IRI, [16] [20]) reveals that individuals vary substantially in the extent to which they are generally predisposed to experience either concern and sympathy (*i*.*e*., *dispositional empathic concern*) or distress and discomfort (i.e., *dispositional personal distress*) in response to other people’s distress [20]. Further, within a specific situation, an observer who is exposed to a target in distress can experience both *situational empathic concern* and *situational personal distress* [16].

Existing research points also to the important role of affective empathy in marital support. First, Devoldre et al. [24] found that dispositional empathic concern and personal distress (i.e., dispositional affective empathy) play a meaningful role in the provision of social support in marriage. Further, the motivation to help is determined by the type of emotion that currently predominates in a person who is exposed to another in need (situational affective empathy) [25][29].

In general, the results of these studies are consistent with Batson’s [25] analysis of the role of emotional empathy in help-giving. In this analysis, an emotional response such as *situational empathic concern* is presumed to be the source of a truly altruistic motivation: the stronger the feelings of compassion for the other person, the greater the motivation to reduce his/her distress. The goal in this case is to alleviate the other person’s distress, rather than one’s own distress. On the other hand, Batson argues that *situational personal distress* can produce helping for largely egoistic reasons. The greater the personal distress the observer experiences, the greater his/her motivation to have it reduced. This self-oriented distress can be reduced either by helping the other or by withholding one’s help and escaping the situation if this is possible. Despite the equivocal nature of this second prediction, the available data support Batson’s [25] claim that the emotions just described indeed predict the level of helping that occurs.

### The present study

In sum, empirical precedents support the importance of *cognitive* as well as *affective* empathy, and of *dispositional* as well as *situational* forms of empathy in predicting providers’ level of spousal support. However, no previous studies have simultaneously included all of these distinct aspects of empathy within a single investigation. Therefore, our goal was to (a) conceptually and empirically differentiate between cognitive and affective forms of empathy and between dispositional and situational forms of empathy, and (b) examine their unique role in actual support transactions in couples. More specifically, in the present study we collected (1) *global self-report measures of cognitive empathy* (i.e., dispositional perspective taking) *and affective empathy* (i.e., dispositional empathic concern and personal distress); (2) *spouses’ interaction-based assessment of cognitive empathy* (i.e., situational perspective taking) *and affective empathy* (i.e., situational empathic concern and personal distress); and (3) *observational measures of support-seeking and support provision behavior*.

From the theory and empirical precedents described above, we predicted that a support provider’s cognitive and affective empathy should assist him/her in navigating the first two stages in Pearlin and McCall’s model [8]. Assuming that empathy has a positive impact during these stages, it should not only promote more positive support but also inhibit the negative forms of support in the final stage of support transactions. With regard to the empathy-relevant traits and reactions that we use as predictors of spousal support in the present study, we propose two general hypotheses and two research questions:

#### Hypotheses and research questions

First, we expect *cognitive empathy* to be associated with greater social support, such that when the support provider takes the perspective of the support seeker and more accurately infers his/her thoughts and feelings, the provider will offer higher levels of positive support (i.e., more emotional and instrumental support), but lower levels of negative support to their support-seeking partner (*Hypothesis 1)*. Second, we also expect *affective empathy* to be associated with spousal support provision, such that when the provider experiences affective empathy (i.e., empathic concern, personal distress), the provider will offer higher levels of positive support (i.e., more emotional and instrumental support) and lower levels of negative forms of support *(Hypothesis 2)*.

In the present study, measures of both dispositional and situational empathy were included. Because previous studies do not provide a basis for making empirically-based predictions about the relative predictive power of the more distal and dispositional empathy measures in contrast to the more proximal and situational ones, we left it up to the data to educate us about the differential impact of dispositional and situational forms of empathy on spousal support provision (*Research Question 1*). Finally, we wanted to explore potential gender differences in the relationship between empathy and the provision of social support. In the previous literature, we found no evidence that allowed us to make specific predictions, so we planned to let the data tell us whether the processes under study are different for men and women who are in the support provider role (*Research Question 2)*.

## Materials and Methods

The present data were collected within a broader observational study on social support in couples; some results of this study—unrelated to the present research questions—appear in [30].

### Ethics statement

The study was approved by the ethical committee of the Faculty of Psychology and Educational Sciences of Ghent University, Belgium.

### Participants

The sample consisted of 50 married/cohabitating couples who were recruited by research assistants from the geographic vicinity of our research centre. The screening criteria stipulated that couples had to have been living together for at least 6 months and have a minimum relationship length of 1 year. The mean ages for the men and the women were 41.10 (*SD* = 14.62, range: 22–76), and 39.63 (*SD* = 15.28, range: 20–77), respectively. The mean duration of couples’ cohabitation/marriage was 16.54 years (*SD* = 14.65, range: 1–55).

### Procedure

After providing their written informed consent, both partners independently completed an online battery of questionnaires. Couples who completed the questionnaires were then scheduled to attend a laboratory session. In this session, the members of each couple participated in two 10-minute videotaped support interaction tasks, each followed by a post-interaction video-review task that enabled an assessment of the support provider’s situational empathic reactions. At the end, the couples were fully debriefed and received a gift voucher of 20 euros.

#### Questionnaires

Relationship satisfaction was assessed with the Dyadic Adjustment Scale (DAS [31]). The mean values of the global DAS within our study were 113.34 for the men (α = .89) and 115.89 for the women (α = .90). DAS norms indicate an average satisfaction score of 114/115 for a married sample, thereby suggesting that our sample is comparable to an average group of married couples in terms of relationship satisfaction [31].

Individual differences in dispositional empathy were assessed by the 28-item Interpersonal Reactivity Index (IRI [20][32]). The *Perspective Taking* subscale measures the cognitive tendency to adopt another’s psychological perspective (7 items; “I try to look at everybody’s side of a disagreement before I make a decision”). The *Empathic Concern* subscale assesses the tendency to experience feelings of warmth, sympathy and concern towards others (7 items; “When I see someone taken advantage of, I feel kind of protective towards them”). Last, the *Personal Distress* subscale measures feelings of discomfort and distress when witnessing others’ negative experiences (7 items; “I sometimes feel helpless when I am in the middle of a very emotional situation”). Each item was rated on a Likert scale (0 = *does not describe me well*, 4 = *describes me very well*). Subscale scores were computed by summing scores for all items included in a specific subscale (αs ranged from .72 to .76).

#### The social support interaction task

The support interaction task we used was similar to the one used in previous observational studies of marital support (e.g., [1][11]). The participants were led into a laboratory that was furnished as a living room and equipped so that the couple’s interaction could be videotaped with their prior knowledge and consent. By random assignment within each dyad, one spouse was designated to be the support seeker and the other spouse to be the support provider. For half of the couples in the first lab discussion, the male partner was designated as the support seeker, with the female partner in the role of the support provider. For the other half of the couples in the first discussion, these roles were reversed. In the second lab discussion, the partners traded their roles so that data could be obtained for both partners in both roles.

Before each discussion, the support seeker was asked to talk to his/her partner about a salient personal problem―defined as any problem the source of which was not the partner or the relationship (e.g., dealing with work stress, changing a bad habit). The partners were told there would be two distinct conversations, one for each partner talking about his/her problem. The partners were allowed to interact up to a maximum time limit of ten minutes.

#### The video review procedure

Immediately after their interaction had been recorded, the partners completed a video-review task (see [33][11]). The partners were asked to imagine living through and re-experiencing their interaction again while they each watched a videotaped copy of the interaction. After each minute of interaction, the videotape was paused to assess each partner’s situation specific empathic reactions. The partners simultaneously completed the video-review task in the same room. However, they were unable to see or talk to one other during the task because they were separated by a wall between them. A research assistant remained present during the review task.

Reports of actual and inferred thoughts and feelings. At each of the “stop points” at which the tape was paused by the experimenter, the participants were instructed to report the content of each of their unexpressed thoughts and feelings at that point in the interaction. The instructions explicitly required the participants to fully report all thoughts and feelings, and to do so as accurately and honestly as possible. In addition, the participants were instructed to make inferences about the unexpressed thoughts and feelings of their partner at that time in the interaction. Specifically, they were required to infer what the partner was thinking or feeling and report the content of this inferred thought or feeling.

Reports of situational empathic concern. At each stop point, we also asked the participants to rate three items inquiring about situational empathic concern (e.g., “At that moment, I was sorry for my partner”) on a 9-point Likert scale (1 = *not at all*, 9 = *very much*). We summed each participant’s responses to these three items and then averaged their responses across the ten stop points to create an index of situational empathic concern (α = .98 for male provider, α = .98 for female provider).

Reports of situational personal distress. We also asked the participants to report their situational arousal and negative affect. Each time the videotape was stopped, the participants were instructed to indicate their current level of arousal (ranging from *totally calm* to *totally nervous*) and their current level of negative affect (ranging from *totally unhappy* to *totally happy*). The participants rated these two items on a 9-point scale. The affect scale was then recoded, and the responses on the two items were summed. These scores were then averaged across the ten stop points to create an index of situational personal distress (α = .95 for male provider, α = .94 for female provider).

#### Observational measures

Observed behaviors of the support seeker and the support provider were coded with the Social Support Interaction Coding System (SSICS, [2]). The support seeker’s behavior could be assigned to one of four categories: positive, negative, neutral, or off-task. The support provider’s behavior could be assigned to one of six categories: positive emotional, positive instrumental, negative, neutral, positive other, or off-task (see [2] for a detailed description). Although the observed support behaviors were coded into 10 categories, we analyzed the data for only the following categories in the current study: (1) *Positive support seeking* (e.g., gives clear analysis of the problem, recognizes partner as an aid, agrees with provider’s suggestions); (2) *Negative support seeking* (e.g., rejects help, criticizes the support provider, makes demands for support, complains); (3) *Positive emotional support provision* (e.g., reassures, encourages expression of feelings, provides genuine encouragement); (4) *Positive instrumental support provision* (e.g., offers specific plan or assistance, gives helpful advice); (5) *Negative support provision* (e.g., criticizes, minimizes problem, is inattentive, offers unhelpful advice).

Two trained observers coded the behavioral data (see [11] for details on coder training). They were told only the topic of the discussion and whose issue was discussed (the male or the female partner’s issue). A randomly selected 20% of the interactions were coded by both observers; all of the inter-observer kappa values indicated good levels of inter-observer reliability (range: .67 to .84). When the coders viewed the videotaped interaction, they assigned codes to each support provider’s speech turn and to each support seeker’s speech turn. Then the proportions of positive seeking, negative seeking, emotional provision, instrumental provision, and negative provision codes were computed by summing each spouse’s total score for a given support category and then dividing that category by the total number of speaking turns. These proportions of support behavior were used as outcome measures in the analyses reported below. The use of proportions is standard practice in research using the SSICS (e.g., [1] [2] [34]).

#### Situational perspective taking

Situational perspective taking (i.e., empathic accuracy) was computed by comparing the written content of each support seeker’s actual thought/feeling entry with that of the corresponding support provider’s inferred thought/feeling entry. Following the recommendations of Ickes, Stinson, Bissonette and Garcia [21], five judges compared each inferred thought/feeling entry with the corresponding actual thought/feeling entry and rated their level of similarity on a 3-point scale ranging from 0 (*essentially different content*) through 1 (*somewhat similar but not the same content*) to 2 (*essentially the same content*). Given the high reliability of the judges’ content accuracy ratings (α = .85), the mean of the empathic accuracy scores rated by the five judges was calculated for each particular inference.

To derive an overall situational perspective taking score for each support provider, the mean empathic accuracy scores were summed across all thought/feeling inferences and then divided by the total number of accuracy points that could be obtained for a given number of inferences, and multiplied by 100 (following this transformation, the range of the situational perspective taking measure was 0 to 100).

### Data analysis

To account for the empirical interdependence of the data (i.e., each partner being involved in the same intimate relationship and the data from each partner resulting from an interaction in which both partners participated [35]), the multi-level Actor-Partner Interdependence Model (APIM [36]) was used. Our data were analyzed using the Generalized Estimating Equations (GEE) approach because this extension of the General Linear Model can empirically account for both positive and negative correlations of the observations within couples.

## Results

### Descriptive statistics

Five couples did not complete the entire research procedure and were excluded from the analyses. All further analyses are based on the results of the 45 remaining couples. Table 1 shows the descriptive statistics.

**Table 1:** Descriptive Statistics.

	Males		Females			
	*M*	*SD*	*M*	*SD*	*t*	*Possible range*
Dispositional empathic concern	15.98	4.22	20.16	3.07	5.37**	0–28
Situational empathic concern	17.98	4.01	15.67	5.27	-2.34*	3–27
Dispositional perspective taking	17.93	3.61	18.09	3.82	0.20	0–28
Situational perspective taking	18.08	14.01	11.76	9.76	-2.34*	0–100
Dispositional personal distress	10.18	3.65	14.91	4.36	5.59**	0–28
Situational personal distress	6.95	2.03	6.68	2.27	-0.60	2–18
Positive support-seeking	63.85	22.68	70.06	16.44	1.49	0–100
Negative support-seeking	2.83	7.51	4.04	9.25	0.68	0–100
Instrumental support provision	16.07	9.55	15.96	13.18	-0.05	0–100
Emotional support provision	8.21	10.68	6.47	7.76	-0.88	0–100
Negative support provision	8.75	12.42	9.24	14.24	0.17	0–100

*Note*.

* *p <* .05

** *p <* .001

As indicated in Table 1, the analysis revealed no significant gender differences in observed support seeking behavior nor in observed support provision behavior (*p*-values between 0.14 and 0.96). The analysis did, however, reveal gender differences favoring men in situational empathic concern (*p* = 0.02) and situational perspective taking (*p* = 0.02). Finally, higher levels of dispositional empathic concern and dispositional personal distress were found in women, as compared to men (*p* < 0.001 and *p* < 0.001, respectively). Correlations between the predictor variables are presented in Table 2.

**Table 2:** Correlations between the Predictor Variables for Males and Females.

	Males	Females
Dispositional empathic concern with dispositional perspective taking	.57**	.31*
Dispositional empathic concern with dispositional personal distress	.06	.03
Dispositional perspective taking with dispositional personal distress	-.33*	-.21
Situational empathic concern with situational perspective taking	.00	.02
Situational empathic concern with situational personal distress	-.34*	.10
Situational perspective taking with situational personal distress	.11	-.07
Dispositional empathic concern with situational empathic concern	.26	.21
Dispositional perspective taking with situational perspective taking	-.08	-.07
Dispositional personal distress with situational personal distress	.00	-.14

*Note*.

* *p <* .05

** *p <* .001

### Data analytic strategy

The data had a two-level structure. All measures of dispositional empathy, situational empathy, and support behaviors were obtained for both partners in each couple. Three separate models were fitted to our data: one for each category of support provision behavior (i.e., emotional, instrumental, and negative support behaviors displayed by the spouse in the support provider’s role). The empathy-related predictors in each model included both dispositional (i.e., IRI scores) and situational ones (i.e., situational measures of perspective taking, empathic concern, and personal distress). For the situational predictors, the support provider’s score, the support seeker’s score, and the interaction with gender were added so that we could model the interdependence of the partners using the APIM. For the dispositional predictors, only the provider’s own score and the interaction with gender was used, as we did not formulate hypotheses about the impact of the support seeker’s dispositional empathy on the provider’s observed support provision. Within each model we also controlled for the potential influence of the support seeker’s and the support provider’s support-seeking behavior (positive and negative) on observed support provision.

The dependent variables were all measured as percentages, as were the measurements of positive and negative support-seeking. The regression weights for the support-seeking behaviors therefore indicate by how many percentages the amount of support provision changes with each percent increase in the support-seeking behaviors.

The empathy measures were centered and scaled. The regression weights for the empathy measures thus indicate by how many percentages the amount of support provision changes with each standard deviation increase in the empathy measures. Gender was effect coded (-1 for male and 1 for female). All main effects are thus averaged over both genders. To get the score for females, the interaction term has to be added to the main effect; to get the score for males, the interaction term has to be subtracted from the main effect. The intercept of the model is an estimate of the percentage of a type of support provision behavior for average partners when no support-seeking behaviors are observed.

### Tests of the research hypotheses

Table 3 presents the final models of the multilevel analyses predicting the support provider’s support provision as a function of the empathy measures (both dispositional and situational), and controlling for support-seeking behaviors.

**Table 3:** Regression Weights and Test Statistics for the Models with Emotional, Instrumental, and Negative Support Provision as Dependent Variable.

	Emotional			Instrumental			Negative		
	*B*	*z*	*p*	*B*	*z*	*p*	*B*	*z*	*p*
Intercept	0.582	0.116	.908	-5.156	-0.762	.446	19.222	4.948	.000**
Gender	3.031	0.642	.521	-6.656	-1.418	.156	-2.193	-0.375	.707
Positive SS	0.109	1.671	.095°	0.212	2.457	.014*	-0.112	-1.220	.222
Positive SS seeker	0.019	0.242	.808	0.094	1.489	.137	-0.050	-0.656	.512
Positive SS x Gender	-0.053	-0.785	.433	0.072	0.742	.458	-0.098	-1.444	.149
Positive SS seeker x Gender	0.021	0.257	.797	0.022	0.315	.753	0.112	2.367	.018*
Negative SS	0.057	0.451	.652	-0.010	-0.056	.955	0.557	5.153	.000**
Negative SS seeker	-0.307	-2.592	.010*	0.307	2.412	.016*	0.645	4.868	.000**
Negative SS x Gender	0.062	0.474	.636	0.195	1.007	.314	-0.133	-1.359	.174
Negative SS seeker x Gender	-0.085	-0.777	.437	-0.108	-0.738	.460	0.272	2.197	.028*
Dispositional EC	-1.035	-0.721	.471	-0.528	-0.355	.722	-3.474	-2.844	.004**
Dispositional EC x Gender	-0.939	-0.761	.447	0.524	0.305	.761	-2.055	-1.470	.142
Situational EC	1.031	0.923	.356	0.854	0.826	.409	-1.603	-1.048	.295
Situational EC seeker	-1.466	-1.779	.075°	0.703	0.495	.621	1.302	1.076	.282
Situational EC x Gender	1.587	1.538	.124	0.320	0.292	.770	2.750	1.687	.092°
Situational EC seeker x Gender	-0.179	-0.207	.836	-0.958	-0.620	.535	0.962	0.627	.531
Dispositional PT	1.248	0.972	.331	-0.948	-0.553	.580	0.202	0.152	.879
Dispositional PT x Gender	-3.685	-2.739	.006**	1.427	0.821	.412	-0.135	-0.094	.925
Situational PT	-0.191	-0.192	.848	2.648	2.168	.030*	-0.364	-0.302	.762
Situational PT seeker	-0.429	-0.389	.697	-1.050	-1.021	.307	0.582	0.521	.603
Situational PT x Gender	-1.477	-1.231	.218	0.400	0.313	.755	2.008	2.114	.035*
Situational PT seeker x Gender	-0.872	-0.677	.498	1.763	1.790	.073°	-1.672	-1.886	.059°
Dispositional PD	-1.238	-1.083	.279	2.749	1.733	.083°	3.132	1.723	.085°
Dispositional PD x Gender	-1.044	-0.727	.467	-0.470	-0.301	.763	-1.109	-0.715	.474
Situational PD	-2.505	-1.827	.068°	1.197	1.307	.191	-0.638	-0.593	.553
Situational PD seeker	1.403	1.466	.143	-2.465	-1.819	.069°	-0.603	-0.645	.519
Situational PD x Gender	0.136	0.105	.916	-2.437	-2.067	.039*	1.262	1.017	.309
Situational PD seeker x Gender	-0.322	-0.393	.694	2.200	1.458	.145	1.222	1.061	.289

*Note*. SS = support seeking, EC = empathic concern, PT = perspective taking, PD = personal distress

° *p* < .10

* *p* < .05

** *p* < .01

#### Value of cognitive (H1) and affective (H2) empathy in explaining emotional support provision

For *emotional support provision*, the analyses revealed a significant interaction of the provider’s dispositional perspective-taking and his or her gender (*β* = -3.68, *p* = .006). For men, higher levels of dispositional perspective-taking were associated with more emotional support provision (*β* = 4.93, *p* = .030). In contrast, for women, the effect was not significant. The provider’s level of emotional support provision was also related to his/her partner’s amount of negative support-seeking, with more negative support-seeking being associated with lower levels of emotional support (*β* = - 0.30, *p* = .010).

#### Value of cognitive (H1) and affective (H2) empathy in explaining instrumental support provision

The provider’s level of *instrumental support provision* was associated with his/her own level of situational perspective-taking. As predicted, providers who displayed greater empathic accuracy provided more instrumental support (*β* = 2.65, *p* = .030). Further, an interaction was found between the provider’s own situational personal distress and gender (*β* = -2.44, *p* = .039). For the male providers, those with higher levels of situational personal distress provided more instrumental support (*β* = 3.63, *p <* .001). For the female providers, the corresponding effect was not significant (*β* = -1.23, *p* = .488). The provider’s level of instrumental support was also related to the support seeker’s level of negative support-seeking, with greater negative support-seeking behavior being associated with more instrumental support (*β* = 0.31, *p* = .016). This effect was complemented by a significant main effect of the support provider’s own level of positive support-seeking (in the seeker role), with more positive support-seeking (in the seeker role) predictive of more instrumental support (in the provider role) (*β* = .21, *p* = .014).

#### Value of cognitive (H1) and affective (H2) empathy in explaining negative support provision

The provider’s dispositional empathic concern had a significant effect on his/her level of *negative support provision*, with higher scores on dispositional empathic concern predicting less negative support provision (*β* = - 3.47, *p* = .004). There was a significant interaction between the provider’s situational perspective-taking and gender (*β* = 2.01, *p* = .035). For male providers, higher scores on situational perspective-taking were associated with providing less negative support (*β* = - 2.37, *p* = .003). This effect was not significant for female providers (*β* = 1.64, *p* = .374). The analyses also revealed a significant interaction effect of the seeker’s negative support-seeking and gender (*β* = 0.27, *p* = .028). More negative support was provided by both men and women in response to more negative support-seeking (*β* = .37, *p* = .037 for men; *β* = .91, *p <* .001 for women); however, this effect was clearly stronger for women. A significant interaction between the seeker’s positive support-seeking and gender was also found (*β* = 0.11, *p* = .018), but for both men and women the simple effect was not significant. Finally, the provider’s negative support provision was related to his/her own level of negative support- seeking (in the seeker role) (*β* = .56, *p <* .001). Providers provided more negative support (in the provider role) when they scored higher on negative support-seeking (in the seeker role).

## Discussion

The present study sought to examine how, after controlling for the support-seeking behaviors of both partners, support providers’ empathic dispositions as well as their situational empathic reactions were related to the provision of spousal support during observed support interactions.

### Summary of results

#### Affective and cognitive empathy

For each of the three types of support provision, the empathy of the support provider—either dispositional or situational—appears to play a meaningful role. However, there seem to be different roles played by *affective and cognitive empathy*, depending on what type of support is examined (*Hypothesis 1 & 2*).

Some types of support provision were related to both the affective and the cognitive components of empathy. As predicted, when providers scored higher on affective empathy (i.e., dispositional empathic concern), they provided lower levels of negative support. In addition, for male partners cognitive empathy (i.e., situational perspective taking in the form of empathic accuracy) played a role in the provision of negative support that was in line with our expectations: scoring higher on cognitive empathy (i.e., situational perspective taking) was related to lower levels of negative support provision. This finding is consistent with the results of previous lab-based studies that have found empathic accuracy to be related to lower levels of behaviorally-coded negative support (e.g. [11]). We interpret this pattern as evidence that efforts to understand one’s partner provide greater insight into the partner’s needs, which in the present study significantly inhibited the male providers from providing unhelpful advice, criticism, etc.

The provision of instrumental support also showed an association with both cognitive and affective empathy. For both partners, higher scores on cognitive empathy (i.e., situational perspective taking) were associated with the provision of positive instrumental support. This finding is also in line with those of previous observational studies that have found situational perspective taking in the form of empathic accuracy to be related to higher levels of behaviorally-coded instrumental support (e.g. [11]). The present replication of this finding supports the previous interpretation that efforts to understand one’s partner provide greater insight into the partner’s specific needs, an insight that facilitates the provision of effective advice, help, and guidance.

For male providers, this kind of support provision also showed a link with affective empathy: higher scores on affective empathy (i.e., situational personal distress) were related to the men’s provision of higher levels of instrumental support to their female partner. One should take into account the possibility that the instrumental support provided here was not necessarily caring in nature. Indeed, from Batson’s [25] perspective, under conditions of difficult escape—such as spousal support interactions—the support provided could have been provided for egoistic (rather than altruistic) reasons. This speculation is consistent with the fact that the men’s level of situational empathic concern was not related to their instrumental support provision.

One type of support provision—emotional support—was uniquely associated with the cognitive component of empathy. As predicted, dispositional perspective taking was related to higher scores on emotional support provision for the male providers. This finding is in line with those of a previous study in which the dispositional tendency to take the perspective of others was found to be positively correlated with self-reports of emotional partner support, although this finding was significant only for the women in that investigation [24].

As a whole, the current findings suggest that, within couples, providers who score higher on the empathy-related measures of (1) tending to take other people’s perspectives, (2) tending to experience either concern and sympathy for other people’s distress, (3) being more successful in “reading” the contents of their partner’s ongoing thoughts and feelings, and (4) feeling something of what their partner feels during the interaction, were the ones who were the most likely to provide the most helpful and least harmful support to their partner.

However, although both affective and cognitive facets of empathy appeared to have value in explaining observed spousal support provision, it should be noted also that a substantial number of our predictions were not confirmed. Out of the twelve expected relationships between *affective empathy* and support, ten were not found in the current study. For *cognitive empathy*, three out of six expected relationships were not confirmed.

Clearly, the null findings applied primarily to the predicted affective empathy-support association. In this regard, two specific issues warrant further discussion. First, despite their intuitive plausibility, none of our predictions about the expected positive relations between situational/dispositional empathic concern and positive spousal support were confirmed. What have previous studies found in this regard? Concerning dispositional empathic concern and self-reports of supportive behaviors, in one study no association was found; in a second study, the predicted pattern was found only for emotional support (in women), and the opposite pattern was found for instrumental support (in men) (see [24]). Finally, in a recently conducted diary study, the link between dispositional empathic concern and positive support was again not found [37]. In summary, the evidence for this intuitively obvious link between dispositional concern and supportive behaviors appears to be weak and equivocal, suggesting the possibility that it is often overridden by the influence of factors that are specific either to the particular support-seeking situation or to the partners’ relationship (i.e., their level of marital satisfaction) [38].

Concerning the role of situational empathic concern in couples’ support provision, to the authors’ knowledge there are no previously published studies on this issue that have examined couples’ observed support interactions. However, in studies on situational empathic concern using other samples (e.g., children), methods (e.g., vignettes), and related outcomes (e.g., tendencies of prosocial behavior) (e.g., [39]), empathy effects have been found. So why were these effects not found in our current support provision study? One possibility is suggested by our previous work documenting a strong association between marital satisfaction and support motivation in couples [13].

This finding suggests that, in general, the motivation to provide support to one’s spouse is determined to a greater extent by how the spouses generally feel about their relationship, than by their individual empathic characteristics or reactions. If so, when compared to the support provision that occurs in the interactions of married couples, situational empathy might be a better motivator of support provision in interactions between strangers who have not yet established strong feelings about the value of their relationship. This possibility is supported by a recent review of the empathy-helping literature [40], which found stronger and more reliable associations between empathic concern and helping for targets who were neither too psychologically close to the helper (e.g., family) nor too far away (e.g., outgroup members).

Another possibility is that it is difficult to find effects of genuine empathic responding on support motivation and provision in a laboratory interaction paradigm because the task demands to provide support are high for both partners. In contrast, self-report studies may be more likely to reveal empathy effects because they typically solicit ratings of global support provision in a format in which perceived demand characteristics are lower.

As a second issue warranting extended discussion, personal distress was not found to be broadly related to observed support, an outcome that was contrary to our prediction that in no-escape situations (like the one used in the current study) personal distress would be associated with higher levels of positive support. However, personal distress was also not found to interfere with social support in the present study; in contrast, the results of at least one previous study found personal distress to be inversely related to prosocial behavior (e.g., [41]). Part of this inconsistency may result from the contradictory effects that personal distress can have. Although it can undoubtedly *motivate* (egoistic) helping, self-oriented distress may also interfere with choosing and enacting the most appropriate forms of support. Thus, the most we can say at this point is that our findings on personal distress contribute to a larger pattern of inconsistent results on the relationship between personal distress and prosocial behavior (see [42]).

#### Dispositional and situational empathy

When we compared how dispositional and situational measures of empathy were associated with spousal support, different results were found *(Research Question 1)*. First, two forms of *dispositional empathy* proved to be related to the provision of spousal support. As described above, for both partners, higher scores on dispositional empathic concern were associated with lower levels of negative support provision to the partner. In addition, male providers who scored higher on dispositional perspective taking provided higher levels of emotional support. Second, two types of *situational empathy* were associated with spousal support provision. As described above, for the affective component of empathy, the situational variant proved to play a role in support provision only for the male provider. When male providers scored higher on situational personal distress, they provided higher levels of instrumental support to their partner. The second variant of situation-specific empathy, situational perspective taking (empathic accuracy), played its predicted role in the provision of both instrumental and negative support (only for for males).

Taken together, both dispositional and situational forms of empathy were associated with support provision, and the pattern was quite similar: three associations were found with situational empathy, whereas two associations were found with dispositional empathy. This finding is in line with Bradbury and Fincham’s contextual model of interaction [43], which holds that features of both the distal (i.e., relatively stable personality variables) and the proximal context (i.e., a person’s subjective reactions elicited by the immediate situation) influence how partners respond to events.

Overall, our findings are consistent with the literature on empathy and prosocial relationship behaviour. Davis [44] recently reviewed the evidence regarding the role of empathy in social relationships, and came to two broad conclusions. First, empathy in all of its forms (dispositional, situational, affective, cognitive) was meaningfully related to a wide variety of social outcomes, including relationship satisfaction, interpersonal conflict, social support, and constructive responses to partner misbehaviour. Second, in almost all of these domains, cognitive forms of empathy (both dispositional perspective taking and situational empathic accuracy) had the stronger effects. This is, of course, exactly the pattern found here; all forms of empathy had some effect on support provisions, but the most reliable findings were for the cognitive forms.

#### Gender

One interesting feature of the current investigation is the degree to which the effects of empathy on spousal support were qualified by gender *(Research Question 2)*. Of the five effects of provider empathy on support behaviour, three took the form of an empathy x gender interaction. Interestingly, all of those interactions revealed the same pattern: empathy had a more pronounced beneficial effect for men than for women. Although the evidence regarding gender differences in the relationship between empathy and observed social support in couples is quite limited, it should be noted that previous laboratory studies that examined the link between situational empathy and spousal support found virtually no gender interactions at all (e.g., [11]).

So what accounts for this surprising pattern? One possibility is the use in the present study of the Actor-Partner Interdependence Model (APIM) to analyse the data; none of the previous lab studies of spousal support employed this model. A second possibility is that in previous lab studies, due to small sample sizes, interactions failed to reach significance. When looking at the broader literature on empathy and prosocial behavior, evidence can be found for empathy being a stronger predictor of prosocial behavior in males than females, in children and adolescents (e.g., [39], [45]).

Regarding the main effects of gender in the present study, women were found to report higher levels of dispositional affective empathy (both empathic concern and personal distress). This pattern is in keeping with the vast majority of research on sex differences in empathy. Women almost uniformly report being significantly higher on the emotional facets of empathy; the same pattern, although weaker, is often found for self-reports of perspective taking ([20] [32]). This weaker gender difference in self-reported perspective taking also characterized our data, in which the wives failed to report higher levels of dispositional perspective taking than their husbands.

Similarly, we did not find women to outperform men in their situational perspective taking (empathic accuracy). In fact, the husbands in the present study achieved higher levels of situational perspective taking than their wives, possibly because the women were more emotionally expressive than their male partners, making the women’s thoughts and feelings easier “to read” (see [46]). As a whole, this pattern of results supports the claim of previous writers that dispositional self-reports of empathic responding are more biased by gender stereotypes than either a performance measure like empathic accuracy or an interaction-based report of empathic responding ([47] [48] [49] [50]). However, it should be noted also that the dispositional measure we used here is more broadly based and the situational measure is measuring empathy in a very specific context---empathy between spouses when talking about salient personal problems as opposed to empathy towards others in general.

With regard to support provision, we found that husbands and wives displayed comparable levels of support provision behaviors, as observed during the two interactions in which they participated. These findings are quite consistent with those reported in previous studies (e.g., [51] [52]), and they indicate that husbands and wives have the same general capability to support each other within their intimate relationship.

In summary, we found gender differences in dispositional affective empathy favoring women, but also found that observed support provision did not differ by gender. This pattern of findings is consistent with the view that gender stereotypes lead women to rate themselves higher on dispositional affective empathy than men do, even though there are no gender differences in the capacity for support provision. In addition, the empathy-support link was found to be stronger in men, suggesting a greater role of empathy in men’s support provision compared to women’s. A similar pattern of findings was reported for empathy and forgiveness by Toussaint and Webb [53]. Based on these authors’ reasoning, one could assume that if empathy is important in men’s support provision, they might be hampered by lower levels of dispositional (affective) empathy, but helped by higher levels of situational (cognitive and affective) empathy, resulting in equal levels of support provision. However, this interpretation is highly speculative and warrants further study.

#### Support-seeking

Our data also point to the *importance of the support-seeking behaviors* of both partners. When the *support seeker* used *negative strategies* (i.e., complaining, criticizing the partner) to seek support, the support provider provided lower levels of emotional support and higher levels of instrumental and negative support. This pattern of results clearly points to the highly interdependent nature of support provision and support seeking behaviors within couple interactions, a finding that has been previously noted in both observational research [34] and diary research [37]. This pattern of results is also in line with reports in the broader couple research literature of the reciprocation of negativity in couples’ interactions (cf. [54]). In the present study, the association between negative support-seeking and negative support provision was stronger for female support providers, suggesting that female providers are especially prone to respond in a more negative way when their male partners use negative strategies to elicit support from them.

Also noteworthy is the fact that spouses who tended to be more negative in eliciting support also tended to show higher levels of negative support provision. The complementary relation was evident for positive support-seeking: when providers elicited support in a positive way, they also provided it in a more positive way—and, specifically, in an instrumental way. This finding suggests that partners tend to behave in similar ways (positively or negatively) when seeking and providing support. Their stylistic similarity across the two roles of support seeker and support provider may point to substantial individual differences in the participants’ communication skills, with impoverished communication skills being reflected in more instances of negative support seeking and negative support provision

### Strengths and limitations

The use of an observational design allowed us to include situational measures of empathy, which enabled us to conduct a more fine-grained exploration of support processes in couples than survey data can provide. In addition, a dyadic approach was used that included data from both the support-seeking and the support-providing spouse. To fully capture support interactions in couples, both partners were observed within both roles. Perhaps the greatest strength of this investigation is the simultaneous inclusion of both dispositional and situational forms of empathy within a single investigation; no previous study has done this. Doing so enabled us to discover that both forms of empathy have unique associations with support provision even when controlling for the influences of the other form. The most important limitation of this research is the small sample size, which reflects the time-consuming and labor-intensive character of an observational design. In addition, we used a sample of white, middle-class couples, which could limit the generalizability of the results. In the future, the use of larger and more heterogeneous samples will be important. For example, it will be valuable to examine these issues with at least a subsample of couples who are currently experiencing high levels of relationship distress.

Finally, it should be noted that the temporal order of the processes under investigation cannot be tested in the present data. As we previously noted [11], the possibility exists that support provision leads to more situational affective and cognitive empathy rather than the other way around (e.g., the support provision of asking how someone feels may help the support giver to accurately infer what the other person is feeling and may give rise to feelings of concern and distress). The usual recommended caution should therefore be exercised in inferring causality from our results, and the issue of causal ordering needs to be addressed in future research using longitudinal designs (e.g., [55]) or experimental designs.

### Conclusion

The findings of this investigation can be interpreted as part of the growing body of evidence regarding the empathy-support link. In the present study, cognitive as well as affective empathy, and dispositional as well as situational forms of empathy played a meaningful role in shaping the provision of spousal support. This nuanced pattern underscores the value of taking a multidimensional approach to the study of both empathy and social support.
